# The impact of time-restricted eating on health-related quality of life: a systematic literature review

**DOI:** 10.1093/nutrit/nuae044

**Published:** 2024-05-10

**Authors:** Brooke E Sones, Brooke L Devlin

**Affiliations:** School of Human Movement and Nutrition Sciences, Faculty of Health and Behavioural Sciences, The University of Queensland, Brisbane, Australia

**Keywords:** chronic disease management, chrono-nutrition, health-related quality of life, quality of life, time-restricted eating

## Abstract

**Context:**

Time-restricted eating (TRE) is a novel dietary intervention shown to facilitate weight loss and improve metabolic health. However, like any dietary intervention, long-term success largely depends on individual adherence, which can be influenced by whether the intervention impacts the individual’s health-related quality of life (HR-QoL). Despite the growing body of research investigating TRE as a dietary approach and its potential impact on HR-QoL in adults, to date there has been no systematic review to summarize these findings.

**Objective:**

To examine the impact of TRE on HR-QoL in adults.

**Data Sources:**

All randomized controlled trials, pre-post and pilot/feasibility studies were searched in PubMed, EMBASE via Ovid, CINAHL, Cochrane Library, and PsycINFO via Ovid until March 20, 2023.

**Data Extraction:**

Two researchers were involved in the screening and paper selection process. A single researcher extracted all relevant data from eligible studies.

**Conclusion:**

Overall, 10 studies were eligible for inclusion in this systematic review. Four studies reported improvements in overall HR-QoL scores among participants with type 2 diabetes, middle-aged women with obesity, generally healthy adults, and generally healthy adult employees. Three studies reported significant and nonsignificant improvements in some domains of HR-QoL assessment tools among overweight, sedentary older adults, overweight or obese adults, and 24-hour shift workers. No studies reported that TRE adversely affected HR-QoL. Improvements in HR-QoL appeared to occur primarily at 12 weeks/3 months. There was no clear relationship between HR-QoL scores and TRE protocol, additional study outcomes, participant health status, age, or adherence. Although further research is required to elucidate the impact of TRE on HR-QoL, the findings reveal that no studies show that TRE adversely affects HR-QoL.

**Systematic Review Registration:**

Open Science Framework (OSF) (The Impact of Time-Restricted Eating on Health-Related Quality of Life: A Systematic Review; https://doi.org/10.17605/OSF.IO/9NK45).

## INTRODUCTION

The incidence of obesity- and diet-related chronic diseases has grown exponentially across the globe and lifestyle interventions (diet and exercise) have been shown to play an integral role in their prevention and treatment.^1–3^ Specifically, energy-restricted diets are commonly used to reduce body weight and improve metabolic health.^4–6^ However, long-term adherence to energy-restricted diets is a consistent barrier to implementation and success.^7–9^ There is a growing body of research investigating the use of time-restricted eating (TRE) as an alternative dietary strategy to traditional energy-restricted diets.^10^

Time-restricted eating is a novel dietary intervention that limits energy intake to a specific window in the day, followed by fasting in subsequent hours.^10^ Thus, TRE shifts the focus from what and how much an individual should eat to when they can eat. The time of the TRE protocol varies, with eating windows typically ranging from 4 to 14 hours, with a common regime being a 16-hour fast and an 8-hour eating window (16:8).^10^ This food-intake window varies from beginning early in the day (early TRE) to late in the day (late TRE).^10^ The goal of TRE is to align dietary patterns with the body’s 24-hour circadian rhythm.^11^ This diurnal clock is an internal mechanism controlled via a central regulator, the suprachiasmatic nucleus in the hypothalamus, and peripheral clock systems found in all cells in the body.^11^ This internal circadian clock has a bidirectional relationship with metabolism and regulates numerous behavioral and physiological processes throughout the body and is influenced via factors in the internal and external environment.^11^ A misalignment in this clock has been associated with numerous metabolic diseases.^10–12^ The timing of energy intake is a primary external factor that influences the alignment of peripheral and central clock systems.^11^ Thus, eating patterns can positively or negatively alter metabolic pathways, and subsequent behavioral and physiological processes. As a result, studies investigating role of TRE in realigning this clock and eliciting beneficial metabolic and physiological health benefits have become increasingly popular.^11^

Research has shown that TRE has the potential to elicit beneficial effects on metabolic and overall health.^10–12^ More specifically, research has explored the effects of TRE on factors including weight management, sleep quality, metabolic risk factors for disease (eg, lipid profile and insulin sensitivity), and management of metabolic diseases.^10–12^ Time-restricted eating has been shown to elicit weight loss and improve metabolic health through the reduction in eating windows, naturally causing individuals to consume less energy.^10–12^ Nonetheless, improvements in metabolic and health outcomes have also been shown to occur in TRE in the absence of an energy deficit.^12^^,^^13^ However, the long-term success of dietary interventions largely depends on an individual’s long-term adherence to the diet, whereby longer-term adherence allows for greater changes and outcomes to occur.^14^^,^^15^ Long-term adherence to dietary interventions has been shown to be influenced by many factors, including an individual's baseline health status, autonomy in dietary interventions, weight loss, the diet’s interference with social occasions, and the influence on the individual’s health-related quality of life (HR-QoL).^15–19^ Improvements in HR-QoL may promote adherence to dietary interventions, as an individual may perceive the dietary pattern as beneficial to their physical and social well-being.^18^

Quality of life (QoL) is a ubiquitous, multidimensional concept that can be assessed relative to disease (ie, disease-specific QoL) or concerning overall health status, irrespective of disease (ie, generic HR-QoL). Compared with disease-specific QoL, HR-QoL is relevant across different diseases and comorbidities.^20^ Generic HR-QoL typically encompasses five dimensions of health, including physical, social, material, and emotional well-being as well as development and activity.^21^ Common validated assessment tools used in the literature include the 12-item Short Form survey (SF-12),^22^ 36-item Short Form survey (SF-36),^23^ EuroQOL 5-Dimension Questionnaire (EQ-5D),^24^ Assessment of Quality of Life–8 Dimensions (AQoL-8D),^25^ the World Health Organization (WHO) Quality of Life–Bref questionnaire (WHOQOL-Bref),^26^ and the 5-item WHO Well-being Index (WHO-5).^27^ Health-related QoL is also influenced by various factors, including an individual's age, body mass index (BMI), baseline health status, changes in body weight, dietary interventions, and inflammatory markers.^18^^,^^28–31^

There is a growing body of research investigating the impact of TRE in adults with varying health conditions on HR-QoL. Despite this, to date there has been no systematic review that synthesizes their results and indicates the overall impact of TRE interventions on HR-QoL.^32–34^ This is integral as TRE is a relatively new dietary intervention that places temporal restrictions on an individual’s diet, rather than what or how much they should be consuming.^10^ Thus, it is plausible that if the timing of the TRE protocol interferes with an individual's social life or everyday activities, this may negatively impact their HR-QoL. In contrast, as TRE interventions do not tend to have strict limitations on the types or amounts of foods consumed, compared with other dietary interventions, this may improve HR-QoL. If TRE is shown to improve HR-QoL in individuals, this, in turn, may enhance their adherence to this dietary intervention. Long-term adherence to TRE may effectively allow for more meaningful health and metabolic outcomes associated with TRE to occur.^10–13^ This review aims to examine the effects of TRE on HR-QOL in adults.

## METHODS

### Study design

This systematic review was conducted in accordance with 2020 Preferred Reporting Items for Systematic Reviews and Meta-Analyses (PRISMA) guidelines (see Appendix S1 in the Supporting Information online).^35^ Prior to its completion the study protocol was registered with Open Science Framework (OSF).^36^

### Eligibility criteria

The inclusion criteria were established using the Population, Intervention, Comparator, Outcome, and Study design (PICOS) principle, and are shown in Table 1.^37^ Studies were eligible if they met the following criteria; adults (≥18 y), with or without chronic disease; TRE as a primary intervention, irrespective of the protocol used; used a validated tool to assess generic HR-QoL as an outcome, irrespective of the assessment tool; and all originally published randomized controlled trials (RCTs), pre-post trials, prospective cohort, and pilot/feasibility studies. Research excluded from the review included animal or in vitro studies, those with participants under the age of 18, non-original published research, and studies that exclusively investigated intermittent, periodic, or religious fasting.

**Table 1:** PICOS criteria for inclusion of studies

Parameter	Criteria
Population	Adults (human) aged ≥18 years, with or without chronic diseases
Intervention	TRE as a primary intervention, irrespective of the protocol used
Control/comparator	With or without a control/comparator group
Outcomes	HR-QoL, irrespective of the tool used
Study design	All originally published RCTs, pre-post trials, prospective cohort, and pilot/feasibility studies

*Abbreviations*: HR-QoL, health-related quality of life; RCT, randomized controlled trial; TRE, time-restricted eating.

### Search strategy

The search criteria were established with assistance from, and approved by, a librarian at the University of Queensland. Primary concepts for the search criteria included “time-restricted eating” and “quality of life,” which were then mapped for key words and control terms (ie, MeSH [Medical Subject Heading] and Emtree terms). The final search was conducted between February 2023 and March 2023, and included “time-restricted eating” or “time-restricted feeding,” and “quality of life” or “well-being” as the primary concepts. A literature search was conducted in the following databases: (1) PubMed, (2) EMBASE via Ovid, (3) CINAHL, (4) Cochrane Library, and (5) PsycINFO via Ovid (B.E.S.). No restrictions on publication date or languages were applied. All publications were then collated using EndNote X9 software (Clarivate, Philadelphia, PA), where duplicates were identified and culled (B.E.S.).^38^ After the selection process, all eligible articles were forward-searched, and their reference lists screened to identify any studies missed in the initial literature search (B.E.S.).

### Selection process

All remaining studies were imported into Covidence and underwent an abstract screening from two researchers (B.E.S., B.L.D.) to identify all potentially eligible papers.^39^ This was followed by a full-text screening by the same two researchers to determine the research used in data extraction. Papers that were deemed eligible for inclusion in the review were based on the predetermined inclusion and exclusion criteria. No conflicts arose; thus, discussion with a third party was not required. All papers published before March 20, 2023, that met the eligibility criteria were included.

### Data collection process

Data from all eligible studies were independently extracted by a single researcher using an adapted template in Covidence and Microsoft Word (Microsoft Corporation, Redmond, WA, USA).^39^ The data collated from each study included information on the subject population (ie, number of participants, age, sex, cultural background, health status), study design (ie, type, length of intervention, tools used to measure HR-QoL, time points of data collection and analysis, additional controls, and interventions implemented), outcomes (comparator, HR-QoL, adherence), and additional parameters that affect bias (funding, number of study reviewers). Data were then refined for further analysis.

### Quality assessment

Each study was assessed for risk of bias, using the American Dietetic Association (ADA) Quality Criteria Checklist.^40^ This tool was selected for its specificity to dietetics research and applicability to both nonrandomized and randomized trials. It assesses studies’ relevance and validity through a series of 4 and 10 questions, respectively, to give the study an overall rating of either “minus/negative (–),” “neutral (∅),” or “plus/positive (+).”^40^ Each question can be given a ranking of “yes,” “no,” “unclear,” or “N/A.” If any of the relevance questions are answered “no,” “unclear,” or “N/A,” the study cannot receive an overall rating of “plus/positive (+).” The study can only be deemed “plus/positive (+)” if all relevance questions are answered “yes” and most of the validity questions (including questions 2, 3, 6, and 7) are answered “yes.”^40^ Each quality assessment was cross-checked and completed by two researchers, and any conflicts were resolved.

### Data synthesis

A systematic review of the data and the results extracted from the included studies was completed. No meta-analysis was conducted. In this review the overall impact of TRE on HR-QoL in each study was discussed, as well as how HR-QoL outcomes may have been influenced by parameters such as population, additional outcomes, duration of intervention, TRE protocol used, and co-interventions.

## RESULTS

### Literature search results

**Figure 1:**
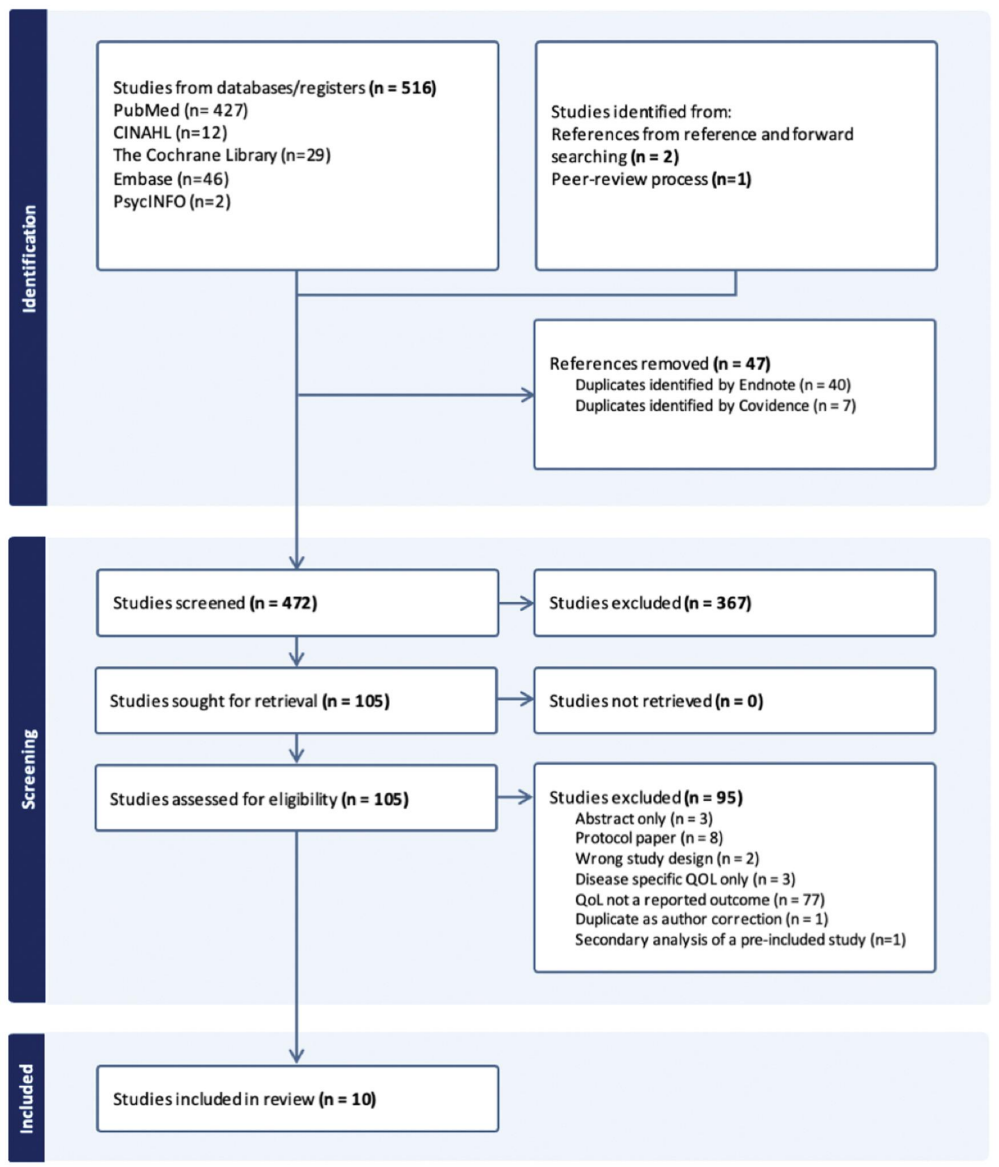
**Flow diagram of the literature search**  **process**. *Abbreviations*: CINAHL, Cumulative Index to Nursing and Allied Health Literature; QoL/QOL, quality of life.

The results of the literature search are shown in Fig. 1. The initial literature search retrieved 516 studies, of which 40 duplicates were removed via Endnote software and seven via Covidence. A total of 469 studies underwent abstract screening, which resulted in the exclusion of 367 papers. From there, 102 studies remained and underwent a full-text screening, 95 of which were excluded based on the pre-established inclusion/exclusion criteria. Reasons for exclusion included abstracts, protocol papers, no reporting of HR-QoL, disease-specific QoL only, duplicate papers as author corrections, and secondary analysis papers of previously included studies. Overall, 7 studies were identified for inclusion in the systematic review based on the initial literature search. From there, the reference list screening and forward searching of included papers identified two additional research papers eligible for inclusion. Finally, through the peer review process, one additional study was identified that met the inclusion criteria. Thus, 10 studies were eligible for data extraction and analysis and included in the systematic review.^32^^,^^34^^,^^41–48^

### Characteristics of included studies

Characteristics of the included studies are shown in Table 2.^32^^,^^34^^,^^41–48^ From the 10 studies there were a total of 511 participants (range: n = 9 to n = 137).^32^^,^^34^^,^^41–48^ The mean (and standard deviation) BMI of participants ranged from 25.5 kg/m^2^ (5.8)^48^ to 34.1 (3.3) kg/m^2^,^45^ and the mean age of participants ranged from 32.3 (10.5) to 77.1 years.^32^^,^^34^^,^^41–47^ While Anic et al^48^ did not report the mean age of participants, 56.7% of participants were aged between 20 and 29 years. The study designs included three RCTs,^32^^,^^41^^,^^42^ one randomized controlled feasibility study,^43^ one pre-post, nonrandomized controlled feasibility study,^44^ one nonrandomized controlled clinical trial,^34^ three pilot pre/post studies,^45–47^ and one prospective cohort study.^48^ There were four studies that included overweight or obese adults,^32^^,^^34^^,^^42^^,^^45^ of which one included middle-aged females,^34^ one in sedentary older adults aged 65 years or older,^45^ and another in patients with obesity and nonalcoholic fatty liver disease (NAFLD).^42^ Two studies included patients with type 2 diabetes (T2D),^41^^,^^44^ one included 24-hour shift workers from San Diego Fire and Rescue,^43^ and three included generally healthy adults^46–48^ (one exclusively looking at healthy adult employees at Ulm University and Hospital).^46^ The 16:8 TRE protocol was the most common, with seven of nine of the studies utilizing this protocol.^32^^,^^34^^,^^42^^,^^45–48^ However, time of the day and whether researchers provided participants autonomy in the protocol timing varied between studies.^32^^,^^34^^,^^41–48^ Two of the studies also included additional interventions, such as the Mediterranean diet and calorie restriction.^42^^,^^47^ Seven studies provided participants with some autonomy in terms of TRE protocols.^32^^,^^43–48^ The tools utilized to assess participants HR-QoL included the SF-12 (n = 3),^41^^,^^42^^,^^45^ SF-36 (n = 4),^32^^,^^43^^,^^47^^,^^48^ EQ-5D (n = 1),^46^ WHOQOL-Bref (n = 1),^34^ AQoL-8D (n = 1),^44^ and WHO-5 (n = 1).^48^ One study utilized two HR-QoL tools, including the WHO-5 questionnaire to measure short-term changes (4 wk) and the SF-36 tool to measure long-term changes (3 mo).^48^ Of the included studies, two were conducted in China,^41^^,^^42^ five in the United States,^32^^,^^34^^,^^43^^,^^45^^,^^47^ two in Germany,^46^^,^^48^ and one in Australia.^44^

**Table 2:** Characteristics of included studies

Reference	Study design	Study population	Age (years); BMI (kg/m^2^)	Sample size	Duration	Intervention: TRE protocol	Control or co-intervention	Adherence: measure; classification of adherence
SF-12								
Che et al (2021) (China)^41^	RCT	Overweight adults with T2D, 18-70 y	Mean age (SD): -TRE: 48.1 (9.82) -C: 48.78 (9.56) Mean BMI (SD): -TRE: 26.42 (1.96) -C: 26.08 (2.14)	120; n = 16 withdrawals, 104 completed study -Females: n = 55 -Males: n = 65 -TRE: 54 -C: 50	12 wk; + 2-wk baseline weight stabilization period	TRE: 14-h fast, 10-h eating window; 08:00-18:00; nil calorie restrictions in eating window; allowed water or tea with no calories in fasting window	C = maintain normal diet; nil co-intervention	Daily log of first and last mealtimes; adherence determined by number of days participant fasted for 10 h
Wei et al (2023) (China)^42^	RCT	Patients with obesity and NAFLD at Nanfang Hospital in Guangzhou, China, aged 18-75 y, BMI 28-45 kg/m^2^	Mean age (SD): -TRE: 32.3 (10.5) -C: 31.7 (8.3) Mean BMI (SD): -TRE: 32.2 (3.4) -C: 32.2 (3.2)	88; n = 14 withdrawals. 81 completed 6 mo, 74 completed 12 mo -Females: n = 39 -Males: n = 49 -TRE: n = 45 -C: n = 43	12 mo	TRE: 16-h fast, 8-h eating window; 08:00–16:00; noncaloric beverages permitted during window	C= nil time restriction on eating; follow same kcal/macronutrient distribution as TRE -Male participants: 1500-1800 kcal/day -Female participants: 1200-1500 kcal/day 40%-55% CHO, 15%-20% Pro, 20-30% fats Provided 1 protein shake/day for 6 mo	Daily log, food pictures, and interviews with trained nutritionists. Adherent if complied to eating window and daily calorie intake.
Anton et al (2019) (USA)^45^	Pilot study	Overweight, sedentary older adults (≥65 y)	Mean age: 77.1 Mean BMI (SD): 34.1 (3.3)	10; n = 1 withdrawals -Females: n = 6 -Males: n = 4 -TRE: n = 10	4 wk; days 1-3 fast 12-14 h, days 4-6 fast 14-6 h, days 7-28 fast 16 h	TRE: 16-h fast (14-18-h target; 15.8-h mean), 8-h eating window; nil dietary restrictions; calorie-free beverage in fast	Nil control; nil co-intervention	Food diaries. Adherent if fasted for 14-18 h per day in weeks 2-4.
SF-36								
Crose et al (2021) (USA)^32^	RCT	Overweight or obese adults, 18- 65 y; BMI ≥ 25 kg/m^2^	Mean age (SD): -TRE: 46.5 (12.2) -C: 44.2 (12.3) Mean BMI: 34.1	20 completed the study. -Females: n = 17 -Males: n = 3 -TRE: n = 11 -C: n = 9	12 wk	TRE: 16-h fast, 8-h eating window; self-selected; allowed water and medication in fasting period; nil restriction of food intake during eating window; average TRE 9.9 h (2.0); average C 15.1 h (1.1)	C = eat ad libitum per usual intake; nil co-intervention	Food diaries in mCC mobile application. Adherent if energy intake was within ±15, ±30, and ±60 min.
Manoogian et al (2022) (USA)^43^	Randomized controlled, feasibility study	24-h shift workers with San Diego Fire and Rescue, aged 21-65 y	Mean age (SD): -SOC: 39.61 (9.39) -TRE: 41.07 (8.71) BMI ≥ 30 kg/m^2^; n (%): TRE: 20 (29)	153; n= 14 withdrawals;137 completed the study -Females: n = 12 -Males: n = 125 -TRE: n = 70 -SOC: n = 67	12 wk	TRE and Mediterranean diet: 14-h fast, 10-h eating window; self-selected: 09:00–19:00 (60% CHO, 25% fat, 15% protein); average 11.3 h eating window (95% CI: 10.73–11.54 h; *P* = 3.29E-17)	Nil control; SOC: Med diet (60% CHO, 25% fat, 15% Pro); average 13.35-h eating window (95% CI: 12.89–13.82 h)	Food diaries in mCC mobile application. Adherence determined by percentage of days logged as not eating in the 10-h fasting window.
Bains et al (2021) (USA)^47^	Pilot pre-post study	Healthy individuals aged 18-65 y, who have never attempted 16:8 TRE	Mean age (SD): 34.0 (11.7) Mean BMI (SD): 27.4 (1.3)	22; n = 6 withdrawals -Females: n = 7 -Males: n = 9 -TRE: n = 16	4 wk	TRE: 16-h fast, 8-h eating window; unrestricted to time of day; nil restrictions on intake; permitted water, black tea/coffee, zero-calorie drinks during fasting	Nil control; nil co-intervention	Self-reported daily journal
AQoL-8D								
Parr et al (2020) (Australia)^44^	Pre-post, nonrandomized controlled, feasibility study	Individuals with T2D aged 35-65 y, BMI 25-45 kg/m^2^	Mean age (SD): 50.2 (8.8) Mean BMI (SD): 34.4 (4.8)	20; n = 1 excluded for lack of data; 19 included in analysis -Females: n = 10 -Males: n = 9 -TRE: n = 19	4 wk; +2 wk habitual baseline period	TRE: 15-h fast, 9-h eating window, 10:00-19:00, on as many days of the week as possible; nil dietary advice provided; nil caffeine during fasting window	Nil control; nil co-intervention	Daily self-reporting (yes/no) and time stamps on food and drink intake records (paper based or EasyDietDiary phone app, Xyris Software, Australia)
WHOQOL-Bref questionnaire								
Schroder et al (2021) (USA)^34^	Nonrandomized controlled clinical trial	Obese middle-aged women, BMI ≥ 30 kg/m^2^	Mean age: -TRE: 36.6 (1.6) -C: 42.3 (3.5) Mean BMI (SD): -TRE: 32.53 (1.13) -C: 34.55 (1.20)	40; n = 8 withdrawals;32 completed the study -Females: n = 32 -Males: n = 0 -TRE: n = 20 -C: n = 12	3 mo	TRE: 16-h fast, 8-h eating window, 12:00-20:00; nil restrictions during eating window	C = maintain same dietary patterns and living habits; nil co-intervention	Not measured
EQ-5D								
Kesztyüs et al (2021) (Germany)^46^	Pilot, pre/post study	Healthy adult employees from Ulm University and Hospital	Mean age (SD): -Females: 47.8 (10.5) -Males: 47.9 (13.5) Mean BMI (SD): -Females: 25.9 (4.8) -Males: 27.5 (3.5)	63; n = 4 withdrawals -Females: n = 54 -Males: n = 9 -TRE: n = 63	3 mo	TRE: 15-16-h fast, 8-9-h eating window; nil limit on food intake during eating window; nil set guideline for time of eating window	Nil control; nil Co-intervention	Documenting of first and last meal
WHO-5 questionnaire (short-term HR-QoL) and SF-36 (long-term HR-QoL)								
Anic et al (2022) (Germany)^48^	Prospective cohort study	Predominantly healthy adults with no specific health profile, aged 18- 59 y	Age: 56.7% between 20 and 29 y BMI range: 18-46.2 kg/m^2^	35: n = 5 withdrawals;n = 30 completed -Females: n = 32 -Males: n = 3	3 mo	TRE: 16-h fast and 8-h eating window on at least 5 days per week; days selected by participants; clear broth, diet drinks, and black coffee allowed during fasting period; nil diet restrictions during eating window	Nil control; nil co-intervention	Food diary created specifically for the trial

*Abbreviations*: AQoL-8D, Assessment of Quality of Life–8 Dimensions; BMI, body mass index; C, control; CHO, carbohydrate; CI, confidence interval; EQ-5D, EuroQOL 5-Dimension Questionnaire; HR-QoL, health-related quality of life; mCC, MyCircadian Clock; MyEQ-5D, 5-Dimension Health-Related Quality of Life Assessment Tool; NAFLD, nonalcoholic fatty liver disease; Pro, protein; RCT, randomized controlled trial; SD, standard deviation; SF-12, 12-item Short Form Survey; SF-36, 36-item Short Form Survey; SOC, standard of care; T2D, type 2 diabetes; TRE, time-restricted eating; WHO-5, 5-item World Health Organization Well-being Index; WHOQOL-Bref, World Health Organization Quality of Life–Bref questionnaire.

### Risk-of-bias assessments

Assessments for risk of bias using the ADA Quality Checklist are outlined in Table 3.^32^^,^^34^^,^^41–48^ One study had an overall plus/positive (+) rating, whereas six studies received a neutral (∅) risk-of-bias rating and three were given negative/minus (–) rating overall.

**Table 3:** Risk-of-bias results using the ADA Quality Checklist from 2 researchers

	Relevance questions				Validity questions										Overall rating
Reference	Q1	Q2	Q3	Q4	Q1	Q2	Q3	Q4	Q5	Q6	Q7	Q8	Q9	Q10	
Anton et al (2019)^45^	Y	Y	Y	Y	Y	Y	N/A	Y	N	N	N	N	Y	Y	∅
Che et al (2021)^41^	Y	Y	Y	Y	Y	Y	Y	Y	Y	Y	Y	Y	Y	Y	+
Crose et al (2021)^32^	Y	Y	Y	Y	Y	U	U	U	N	Y	Y	N	Y	N	–
Kesztyüs et al (2021)^46^	Y	Y	Y	Y	Y	N	N/A	Y	N	Y	N	N	Y	Y	∅
Manoogian et al (2022)^43^	Y	Y	Y	Y	Y	N	Y	Y	U	Y	N	N	Y	N	∅
Parr et al (2020)^44^	Y	Y	Y	Y	Y	Y	N/A	Y	N	N	N	Y	Y	Y	∅
Schroder et al (2021)^34^	Y	Y	Y	Y	Y	Y	N	N	N	N	N	N	Y	Y	–
Wei et al (2023)^42^	Y	Y	Y	Y	Y	N	Y	Y	Y	Y	Y	N	N	Y	∅
Bains et al (2021)^47^	Y	Y	Y	Y	Y	N	N/A	Y	N	N	N	N	Y	N	–
Anic et al (2022)^48^	Y	Y	Y	Y	Y	U	N/A	Y	N	U	Y	N	Y	Y	∅

*Abbreviations*: ADA, American Dietetic Association; N, no; N/A, not applicable; U, unclear; Y, yes; –, negative/minus; ∅, neutral; +, plus/positive.

### Results of included studies

Results from studies included in this review are outlined in Table 4.^32^^,^^34^^,^^41–48^

**Table 4:** Results, adherence, and limitations of included studies

Reference	Effect on HR-QoL	Reported adherence (ie, days adherent or mean as a percentage)
Adults with overweight/obesity		
Anton et al (2019)^45^	SF-12 scores Baseline [mean (SD)]; post-intervention [mean (SD)]; Cohen’s *d* [effect size, (*P* value)]: -Physical function: [12.6 (3.1)]; [14.9 (2.0)]; [0.52 (0.188)] -Mental function: [22.0 (2.1)]; [22.8 (1.7)]; [0.41 (0.285)] -Total score: [35.6 (4.6)]; [37.7 (3.2)]; [0.54 (0.170)]	Mean adherence, 84%
Crose et al (2021)^32^	SF-36 scores Baseline [mean (SD) (range)]; post-intervention [mean (SD) (range)]; *P* values (group effect, time effect, group × time interaction): -Physical function: [89.1 (10.7) (70-100)]; [91.4 (11.6) (65–100)]; (0.32, 0.27, 0.91) -Role limitations due to physical functioning: [88.6 (30.3) (0-100)]; [93.2 (22.6) (25–100)]; (0.31, 0.03, 0.20) -Bodily pain: [79.8 (15.3) (45–100)]; [85.5 (18.5) (45–100)]; (0.84, 0.19, 0.47) -General health: [66.4 (22.7) (5–90)]; [69.5 (57.5) (30–90)]; (0.86, 0.58, 0.58) -Energy: [57.7 (7.5) (45–70)]; [51.8 (8.1) (45–65)]; (0.27, 0.15, 0.32) -Social function: [79.5 (32.7) (0–100)]; [97.7 (5.1) (87.5–100)]; (0.53, 0.03, 0.32) -Role limitations due to emotional health: [66.7 (42.2) (0–100)]; [97.0 (10.0) (66.7–100)]; (0.15, 0.21, 0.04) -Mental health: [66.2 (8.3) (56–84)]; [68.7 (6.4) (60–80)]; (0.32, 0.20, 0.68) -Health transition: [52.3 (23.6) (0–100)]; [68.2 (4.9) (50–100)]; (0.03, 0.01, 0.05)	Days adherent to ±15 min of the 8-h eating window was 55.5% (SD, 22.4%), ± 30 min was 60% (SD, 23 %), and ±60 min was 66.3% (SD, 20.7%)
Schroder et al (2021)^34^	WHOQOL-Bref questionnaire: Results reported in original paper where presented in figure format with no figures reported. Total WHOQOL significantly improved following TRE and self-perception sub-domain was the only sub-domain with significant improvement.	Not reported
Adults with obesity and nonalcoholic fatty liver disease		
Wei et al (2023)^42^	SF-12 Physical Component summary scores, mean (SD): -TRE baseline: 44.5 (7.0) -TRE 6 months: 47.4 (8.2) -TRE 12 months: 48.0 (8.0) SF-12 Mental Component summary scores, mean (SD): -TRE baseline: 54.0 (7.0) -TRE 6 months: 53.9 (7.7) -TRE 12 months: 52.5 (7.9)	Mean adherence (SD): -6 months, 87.9% (10.3%) -12 months, 85.0% (10.7%)
Adults with type 2 diabetes		
Che et al (2021)^41^	SF-12 scores, TRE group, mean (SD): -Week 0: 63.56 (8.18) -Week 12: 69.48 (7.09) SF-12 scores, control group, mean (SD): -Week 0: 62.78 (7.49) -Week 12: 64.49 (8.87) Mean change (SD) [% change]: -TRE: 5.92 (1.38) [9%] -Control: 1.71 (1.4) [3%] -*P* value < 0.001	Reported “compliance” with the TRE intervention was ‘excellent’, >6 days/wk adherent over 12 wk
Parr et al (2020)^44^	AQoL-8D Habitual—post-TRE (*Z*-statistic, effect size, *P* value): -Independent living: (−0.919, 0.16, 0.37) -Happiness: (0.014, 0.07, 0.99) -Mental health: (0.253, 0.00, 0.08) -Coping: (-0.01, 0.00, 0.92) -Relationship value: (-0.08, 0.00, 0.94) -Self-worth: (1.15, 0.22, 0.27) -Pain value: (-0.10, 0.00, 0.92) -Senses: (-0.14, 0.00, 0.89)	Adherence ranged from 4% to 100%; 43% reported adherent but time stamps revealed they were not
Adults with varying health statuses		
Manoogian et al (2022)^43^	SF-36 scores Baseline [mean (95% CI)]; post-intervention [mean (95% CI)]; changes in SF-36 scores [mean (95% CI), (*P* value); mean change TRE-SOC (V3-V1), (*P* value)]: -Physical functioning: [96.73 (94.92 to 98.55)]; [97.11 (95.80 to 98.42)]; [0.38 (-1.15 to 1.91) (0.623); -2.64 (-5.94 to 0.64) (0.114)] -Role limitations due to physical health: [93.93 (89.34 to 98.52)]; [94.88 (90.96 to 98.80)]; [0.95 (-4.92 to 6.83) (0.747); 2.23 (-7.67 to 12.14), 0.656] -Role limitations due to emotional problems: [95.71 (91.67 to 99.76)]; [93.81 (88.69 to 98.93)]; [-1.90 (-8.24 to 4.43) (0.550); 10.60 (1.04 to 20.16), (0.030*)] -Energy/fatigue: [65.00 (60.97 to 69.03)]; [65.46 (60.85 to 70.08)]; [0.46 (-3.26 to 4.18) (0.804); 3.52 (-2.16 to 9.21), (0.222)] -Emotional well-being: [84.54 (82.04 to 87.05)]; [83.94 (81.53 to 86.35)]; [-0.60 (-2.80 to 1.60) (0.588); 4.46 (0.29 to 8.63), (0.036*)] -Social functioning: [94.25 (91.59 to 96.91)]; [93.43 (90.42 to 96.44)]; [-0.82 (-4.12 to 2.48) (0.621); 2.22 (-2.87 to 7.30), (0.390)] -Pain: [84.43 (81.47 to 87.39)]; [85.14 (81.61 to 88.67)]; [0.71 (-2.97 to 4.40) (0.700); 3.33 (-1.98 to 8.64) (0.217)] -General heath: [79.73 (76.66 to 82.80)]; [78.77 (75.54 to 82.00)]; [-0.96 (-3.39 to 1.46) (0.431); -0.03 (-3.92 to 3.86), 0.988] -Health change: [54.41 (48.89 to 59.94)]; [62.87 (57.69 to 68.04)]; [8.46 (2.58 to 14.33) (0.005**)]; [2.83 (-6.13 to 11.78), 0.533]	Participants were nonadherent on approximately 1–2 days/wk
Generally healthy adults		
Kesztyüs et al (2021)^46^	Post-TRE EQ-5D scores: mean change (SD)^a^ -Females: 6.0 (13.2) -Males: 4.8 (7.3) -Total: 5.8 (12.4) *P* value, 0.008	Fasting goal 15-16 h reached 72.2% ± 18.9%; ranged from 16.9% to 97.7%
Bains et al (2021)^47^	SF-36 scores Baseline [median (min, max)]; post-intervention [median (min, max)]; [*P* value, (*r*)]: -Physical functioning: [3.0 (2.8, 3.0)]; [3.0 (2.8, 3.0)]; [0.16 (0.35)] -Role limitations due to physical health: [2.0 (1.0, 2.0)]; [2.0 (2.0, 2.0)]; [0.07 (0.46)] -Role limitations due to emotional problems: [2.0 (1.0, 2.0)]; [2.0 (1.0, 2.0)]; [0.67 (0.11)] -Energy/fatigue: [3.5 (2.5, 4.8)]; [3.8 (2.5, 4.8)]; [0.53 (0.16)] -Emotional well-being: [4.2 (3.8, 5.2)]; [4.1 (3.8, 4.8)]; [1.0 (0.00)] -Social functioning: [3.0 (2.5, 3.5)]; [3.0 (2.0, 3.5)]; [0.21 (0.32)] -Pain: [1.5 (1.0, 3.0)]; [1.3 (1.0, 2.5)]; [0.23 (0.30)] -General health: [3.0 (2.4, 3.4)]; [2.7 (2.0, 3.2)]; [0.14 (0.37)]	Adherent 24.2 ± 4.0 days out of 28
Anic et al (2022)^48^	WHO-5 questionnaire scores, mean (SD): -Baseline: 15.6 (4.6) -Four weeks: 18.0 (3.6) *-P* value:< 0.001 SF-36 scores (baseline mean ± SD vs post-intervention mean ± SD; *P* value)^b^: Physical health: 92.3 ± 11.6 vs. 96.5 ± 6.3; *P*= 0.015 -Vitality: 54.5 ± 21.2 vs. 65.8 ± 15.5, *P*< 0.001 -Mental health: 75.5 ± 12.0 vs. 81.7 ± 9.0, *P*< 0.001 -Social functioning: 83.9 ± 18.9 vs. 92.9 ± 9.2, *P*= 0.021 -Physical pain: 74.1 ± 31.8 vs. 89.5 ± 14.9; *P*= 0.008 -General health perception: 71.8 ± 15.5 vs. 80.4 ± 13.9, *P*= 0.001	Participants fasted an average of 5.5 days/wk

* *P* < 0.05;

** *P* < 0.01.

a Eleven missing values.

b Nil reporting of raw data for categories with no changes in HR-QoL.

*Abbreviations*: AQoL-8D, Assessment of Quality of Life–8 Dimensions; CI, confidence interval; EQ-5D, EuroQOL 5-Dimension Questionnaire; HR-QoL, health-related quality of life; NAFLD, nonalcoholic fatty liver disease; SD, standard deviation; SF-12, 12-item Short Form Survey; SF-36, 36-item Short Form Survey; SOC, standard of care; TRE, time-restricted eating; WHO-5, 5-item World Health Organization Well-being Index; WHOQOL-Bref, World Health Organization Quality of Life–Bref questionnaire.

#### Improvements in overall HR-QoL

Four studies showed significant increases in overall HR-QoL scores after 12 weeks/3 months of TRE.^34^^,^^41^^,^^46^^,^^48^ These improvements were seen in patients with T2D,^41^ generally healthy adults and adult employees,^46^^,^^48^ and middle-aged women with obesity.^34^ Among middle-aged women with obesity, self-perceived QoL had the greatest increase using the WHOQOL-Bref questionnaire, attributed to a reduction in body weight and waist circumference.^34^ In healthy adult employees, statistically and clinically significantly improvements in EQ-5D scores were reported to be independent of changes in body weight, waist circumference, and fasting intensity (15 to 16 h fasting).^46^ Anic et al^48^ reported improvements in HR-QoL among generally healthy adults after 4 weeks and 3 months of TRE, with improvements after 3 months seen in 6 of 8 of the categories of the SF-36 tool (physical health, vitality, mental health, social functioning, physical pain, and general health perception).^48^ Among patients with T2D, TRE was shown to improve overall SF-12 by 9%, compared to 3% in the control group.^41^ The authors reported that improvements in HR-QoL were specifically seen in “physical function” and “daily activity,” although only overall pre and post SF-12 scores were provided in this study.^41^

#### Some improvements in HR-QoL measures

Three studies showed some significant and nonsignificant changes in some domains/scales within HR-QoL measurement tools after 4 and 12 weeks of TRE.^32^^,^^43^^,^^45^ Improvements were seen in mental and physical domains of SF-12 tools in overweight, older sedentary adults, with the greatest improvement in the “physical health” domain.^45^ In adults with overweight or obesity, there were modest improvements in the SF-36 items “health transition” and “role limitations due to emotional health.” The authors stated that these improvements were independent of weight and eating window (self-selected, 16:8).^32^ Among 24-hour shift workers there was a significant improvement in participants’ “health change” scores using the SF-36 tool, and less of a reduction in “role of limitations due to emotional health” and “emotional well-being” compared with the standard-of-care (SOC) comparator group.^43^

#### No improvements in HR-QoL measures

Three studies showed no changes in overall HR-QoL and reported no changes in any of the tool measures after 4 weeks and 12 months of TRE.^42^^,^^44^^,^^47^ Study populations included individuals with T2D (AQoL-8D),^44^ healthy adults (SF-36),^47^ and patients with obesity and NAFLD (SF-12).^42^ The authors who reported no change among healthy adults attributed this to high baseline scores, which remained high at the conclusion of the study. Maintenance of high baseline HR-QoL scores were attributed to reduced stress, improved body composition, and stabilization of sleep patterns in participants.^47^

#### Population health status and HR-QoL outcomes

Six studies included patients with chronic disease and/or risk factors for chronic disease.^32^^,^^34^^,^^41^^,^^42^^,^^44^^,^^45^ Among the 4 studies that included individuals with overweight and obesity,^32^^,^^34^^,^^42^^,^^45^ one study showed overall improvements in HR-QoL (middle-aged women with obesity) compared with baseline values,^34^ two showed significant and nonsignificant improvements in some domains of HR-QoL tools (adults who were overweight or obese and older overweight sedentary adults) compared with baseline,^32^^,^^45^ and one study showed that TRE did not impact HR-QoL (patients with obesity and NAFLD) compared with the comparator group (calorie-restricted diet).^42^ Of the two studies that included patients with T2D,^41^^,^^44^ one showed that TRE did not impact HR-QoL,^44^ while the other showed that TRE significantly improved HR-QoL, specifically in perceived physical functioning and daily activities scores.^41^ One study included 24-hour shift workers, with and without risk factors for chronic diseases, and showed that TRE improved some domains of HR-QoL compared with the SOC group.^43^ Three studies included generally healthy adults,^46–48^ of which two showed that TRE improved HR-QoL (including among healthy adult employees at Ulm University and Ulm University Hospital),^46^^,^^48^ while one study did not report any significant changes in HR-QoL scores.^47^

#### Population BMI and HR-QoL outcomes

In studies where TRE was shown to improve overall HR-QoL scores,^34^^,^^41^^,^^46^^,^^48^ the mean BMI of participants were 26.42 kg/m^2^ (1.96),^41^ 32.53 kg/m^2^ (1.13),^34^ and 25.5 kg/m^2^ (5.8),^48^ and the sex-specific BMI were 25.9 kg/m^2^ (4.8) for females and 27.5 kg/m^2^ (3.5) for males.^46^ In studies that showed that TRE improved some domains of HR-QoL (significantly and nonsignificantly),^32^^,^^43^^,^^45^ the mean BMI of participants were 34.1 kg/m^2^ (3.3)^45^ and 34.1 kg/m^2 ^(7.5),^32^ with one study that only reported the number of participants with a BMI of  30 kg/m^2^ (n = 20, 29%) or higher.^43^ In studies that reported that TRE did not result in any changes in HR-QoL, the mean BMI of participants were 34.4 kg/m^2^ (4.8),^44^ 32.2 kg/m^2^ (3.4),^42^ and 27.4 kg/m^2^ (1.3).^47^

#### Population age and HR-QoL outcomes

One study included older adults aged 65 years and older, with a mean age of 77.1 years.^45^ The authors found small nonsignificant improvements in mental and physical health domains of QoL.^45^ Two studies investigated the effects of TRE on middle-aged adults, with mean age ranges of 50.2 (8.8)^44^ and 36.6 (1.6) years.^34^ Of these, one study showed no significant changes in HR-QoL,^45^ whereas the other showed that TRE increased HR-QoL scores.^34^

#### Study duration and HR-QoL outcomes

Three studies had a total duration of 4 weeks, and all showed that TRE did not elicit any significant changes in overall HR-QoL outcomes.^44^^,^^45^^,^^47^ Six studies had an intervention period of 12 weeks/3 months,^32^^,^^34^^,^^41^^,^^43^^,^^46^^,^^48^ 4 of which showed that TRE resulted in significant improvements in participants’ overall HR-QoL.^34^^,^^41^^,^^46^^,^^48^ One study had a total intervention period of 12 months and showed that HR-QoL outcomes were similar between both intervention groups at 6 and 12 months.^42^

#### TRE protocol and HR-QoL outcomes

Seven studies incorporated the 16:8 TRE protocol.^32^^,^^34^^,^^42^^,^^45–48^ Overall, two studies showed no improvements in HR-QoL,^42^^,^^47^ three showed significant improvements in overall HR-QoL,^34^^,^^46^^,^^48^ and two showed nonsignificant improvements in some aspects of HR-QoL measures.^32^^,^^45^ Additionally, two studies utilized a 14:10 TRE protocol^41^^,^  ^43^: One study reported a significant improvement in overall HR-QoL,^41^ while the other showed improvements in one area of HR-QoL scores.^43^ However, Parr et al^44^ incorporated a 15:9 TRE protocol and reported no changes in overall HR-QoL outcomes after TRE.

Five studies reported the average start and finish times of eating and fasting windows.^34^^,^^41–44^ Of these, two began the eating window at 8 am,^41^^,^^42^ with one study concluding at 4 pm and showing no change in HR-QoL scores^42^ and the other concluding at 6 pm and showing significant improvements in HR-QoL scores.^41^ Additionally, one study had an eating window from 9 am to 7 pm, and showed that TRE significantly improved “health change” of the SF-36 HR-QoL assessment tool.^43^ Another study had an eating window between 10 am and 7 pm, and showed that TRE did not elicit any change in HR-QoL scores.^44^ Schroder et al^34^ prescribed participants to an eating window from 12 pm to 8 pm and results showed significant improvements in HR-QoL with TRE.

#### Autonomy in TRE protocol and HR-QoL outcomes

In the context of TRE, participant autonomy in the TRE protocol refers to their ability to self-select the fasting and feeding hours, and/or days of the week the TRE protocol is adhered to. One study informed participants to aim for a fasting period of 14–18 hours, with no set start or finish times, and showed small nonsignificant improvements in some HR-QoL domains.^45^ Three studies asked participants to self-select a 16:8 TRE regime,^32^^,^^47^^,^^48^ of which Anic et al^48^ showed overall improvements in HR-QoL, Crose et al^32^ showed nonsignificant improvements in some domains of HR-QoL, and Bains et al^47^ showed no statistically or clinically significant changes in HR-QoL after TRE interventions. Notably, Anic et al^48^ also instructed participants to follow the 16:8 protocol on at least 5 days per week. Another study instructed participants to eat within a self-selected 8–9-hour window and results showed that TRE statistically and clinically significant improved HR-QoL.^46^ Manoogian et al^43^ allowed participants to self-select an eating window at the start of the study, which remained throughout. Participants selected an eating window from 09:00 to 19:00; however, the average fasting period was 11.3 hours. Results showed that there were significant improvements in participants’ perception of HR-QoL.^43^ Parr et al^44^ prescribed participants an eating window between 10:00 and 19:00 and provided them with some autonomy by telling them to adhere to this on “as many days of the week as possible.” Results of this study showed no statistically or clinically significant changes in HR-QoL after 4 weeks.^44^ In contrast, three studies did not provide participants with any autonomy regarding TRE interventions.^34^^,^^41^^,^^42^ Of these studies, 2 showed significant improvements in overall HR-QoL scores,^34^^,^^41^ whereas one study showed no significant changes in HR-QoL compared with comparator groups.^42^

## DISCUSSION

This systematic review examining the impact of TRE interventions on HR-QoL in adults showed that TRE did not appear to negatively impact HR-QoL outcomes.^32^^,^^34^^,^^41–48^ Significantly positive improvements in HR-QoL were shown to predominantly occur in interventions with a duration of 12 weeks/3 months, nonspecific to health status.^34^^,^^41^^,^^46^^,^^48^ However, only one of 10 studies that were included exceeded this duration and nonsignificant improvements in some aspects of HR-QoL were still shown in several studies.^32^^,^^43^^,^^45^ Thus, it could be suggested that TRE may play a role in improving some aspects of HR-QoL and does not appear to adversely affect HR-QoL outcomes. Nonetheless, each study varied in level of adherence, sample population, duration of intervention, design, co-interventions, HR-QoL assessment tool, and TRE protocol used, which may impact HR-QoL outcomes in TRE interventions.^32^^,^^34^^,^^41–48^ Thus, the effect of these variables on HR-QoL outcomes was analyzed to determine if changes, or lack of changes, in HR-QoL after TRE were influenced by additional parameters.

First, it cannot be concluded that effect of TRE on HR-QoL was influenced by the population’s health status. Improvements in HR-QoL outcomes after TRE interventions were seen in both generally healthy adults^46^^,^^48^ and among individuals with or at risk of chronic diseases.^32^^,^^34^^,^^41^^,^^45^ Therefore, the health status of participants does not appear to influence the impact of TRE on HR-QOL. However, five of seven of the studies that showed TRE improved overall,^34^^,^^41^^,^^48^ or some aspects of HR-QoL tools,^32^^,^^45^ included populations at risk of or diagnosed with chronic disease, including T2D, overweight, and obesity.^32^^,^^34^^,^^41^^,^^45^ Research shows that individuals with chronic diseases, including people with overweight, obesity, and T2D, generally have lower HR-QoL compared with generally healthy adults.^28^^,^^29^^,^^49–55^ Thus, they may be more likely to see improvements in HR-QoL after interventions compared with healthy adults due to lower baseline scores. In line with this, Bains et al^47^ reported that the lack of improvements in HR-QoL among generally healthy adults was due to high baseline scores. Therefore, it is likely that greater HR-QoL outcomes occur in TRE interventions among populations with a poorer baseline health status.

Additionally, TRE was also shown to improve (significantly and nonsignificantly) perceived health and physical, emotional aspects of HR-QoL in populations with, or with risk factors for, chronic diseases.^32^^,^^34^^,^^41^^,^^43^^,^^45^ More specifically, this included physical health, physical functioning, daily activities, health transition, perceptions of health and QoL, and role limitations due to emotional health scores of HR-QoL tools.^32^^,^^34^^,^^41^^,^^43^^,^^45^ This is in line with current research, which shows that people with overweight, obesity, and T2D tend to show reductions in perceived health and physical, emotional, and mental components of HR-QoL.^29^^,^^49^^,^^53–55^ This further supports the theory that greater HR-QoL outcomes in TRE interventions occur among populations with a poorer baseline health status, yet further research is required.

Moreover, lower baseline physical and emotional aspects of HR-QoL in these populations have been associated with a higher BMI.^29^^,^^54^^,^^55^ A higher BMI has been shown to negatively affect an individual’s physical functioning, mobility, self-care, pain, fatigue, and day-to-day activities, and increase the risk of anxiety, depression, eating disorders, impaired body image, and low self-esteem.^29^^,^^54^^,^^55^ In effect, improvements in BMI and weight have been shown to improve an individual’s HR-QoL.^56–60^ Studies have also found that the magnitude of the effect of weight loss on HR-QoL is correlated with an individual’s starting BMI, whereby greater improvements in HR-QoL were seen in those with a higher BMI and the most significant improvements seen in perceived physical HR-QoL.^60^ Despite this, with the current limited body of literature available, there was no clear relationship between weight loss, BMI, and HR-QoL in TRE interventions.^32^^,^^34^^,^^41–48^ Nevertheless, not all studies that showed improvements in weight, BMI, and HR-QoL after TRE exclusively investigated the relationship between these outcomes.^41^^,^^43^^,^^45^ Notably, one of the studies that reported changes that in HR-QoL were independent of changes in body composition specifically included generally healthy adults,^46^ whereas Schroder et al^34^ reported that changes in HR-QoL scores among middle-aged women with obesity were due to reductions in waist circumference and body weight. More specifically, the authors stated that improvements in body composition promoted improved body image, increasing participants’ self-perceived QoL.^43^ This is in line with current research that shows that weight loss improves emotional aspects of HR-QoL, body image, and self-esteem.^61–64^

The relationship between improvements in HR-QoL, weight, and BMI may also be associated with improvements in inflammatory cytokines.^31^ Research has reported that individuals with a higher BMI may also have higher levels of inflammatory markers, including C-reactive protein (CRP),^31^^,^^65^ and improvements in BMI have been associated with reduction in inflammatory markers and improved HR-QoL.^31^ Despite this, no study in this review specifically explored the possible relationship between these factors.^32^^,^^34^^,^^41–48^ Manoogian et al^43^ reported some significant improvements in HR-QoL domains, weight, BMI, and CRP levels among those with CRP levels of 2.0 mg/dL or greater at baseline after TRE. However, these improvements in CRP were also seen among individuals in the SOC comparator group, with baseline CRP levels of 2.0 mg/dL or greater at baseline.^43^ Notably, both groups were also instructed to follow the Mediterranean dietary pattern, a dietary pattern associated with a reduction in inflammatory markers.^66–68^

Furthermore, studies that showed improvements in weight, BMI, and HR-QoL also showed improvements in, or less of a reduction in, emotional and physical aspects of QoL.^41^^,^^43^^,^^45^^,^^48^ It could be suggested that improvements in perceived physical health within studies were due to improved physical functioning, which has been correlated with weight loss.^57–63^ For instance, one study showed that TRE improved weight and “perceived physical function” and “physical activity” scores of the SF-12 tool in patients with T2D.^41^ This was despite the participants’ step count remaining similar between the TRE and control groups.^41^ Thus, it could be suggested that reductions in body weight improved perceptions of physical functioning and overall physical components of HR-QoL. Nonetheless, more research is required to investigate the link between these outcomes and changes in HR-QoL in TRE interventions to further understand the impact.

Participants’ age is another factor that may influence HR-QoL outcomes. As only one study investigated the impact of TRE on HR-QoL among older adults (≥65 y),^45^ there is currently not enough research to confirm how the impact of TRE on HR-QoL may vary depending on an individual’s age. Nonetheless, this study showed that TRE improved both physical and mental domains of HR-QoL in older adults, with the greatest improvements seen in perceived physical health, attributed to improvements in perceived physical functioning and ability to perform daily activities.^45^ This is noteworthy as research has shown that older age is often associated with a reduced HR-QoL.^30^^,^^69–71^ More specifically, this has been correlated with an increased risk of chronic disease, increased inflammatory markers, and a decline in physical and mental function due to loss of skeletal muscle mass, strength, mobility, cognitive impairment, and social support.^30^^,^^31^^,^^69–71^ More research is required to investigate the potential differing effects of TRE on HR-QoL between older and younger adults.

Results of studies included in this review indicate that intervention periods of 12 weeks/3 months may elicit more of an impact on HR-QoL outcomes.^32^^,^^34^^,^^41^^,^^43^^,^^46^^,^^48^ Importantly, there was also a lack of studies that had a duration of greater than 12 weeks.^32^^,^^34^^,^^41^^,^^43–48^ It is also likely that TRE interventions that run for a shorter duration (4 wk) are not long enough to elicit significant changes in an individual’s HR-QoL. Notably, the studies in this review with a duration of 4 weeks were pilot/feasibility studies,^44^^,^^45^^,^^47^ which often lack the power to determine meaningful changes.^72^ However, it may not be unlikely for changes in HR-QoL to occur after shorter durations of adherence to TRE, as Anic et al^48^ showed that TRE significantly improved HR-QoL in some participants after 4 weeks. Nonetheless, there is currently not enough evidence to determine the long-term effects of TRE on HR-QoL. A recent systematic scoping review reported that adherence to TRE interventions was reduced with longer study durations, whereby adherence to TRE was negatively impacted by factors including provision of support, autonomy, and the TRE protocol.^73^

Within the literature the TRE protocol varies, as eating and fasting windows can vary in length and time of day.^10^ The results of studies included in this review indicated that there was no clear relationship between HR-QoL outcomes and the time or duration of the TRE protocol.^32^^,^^34^^,^^41–48^ Due to the current limited body of research available, the possibility that this may influence HR-QoL outcomes cannot be dismissed. Research shows that, if TRE protocols interfere with an individual’s social eating and drinking occasions, this negatively impacts their participation.^19^ Thus, it is plausible that, if the TRE protocol interferes with an individual’s daily routine, this may, in turn, negatively impact HR-QoL outcomes. For instance, if the individual is a habitual morning person, later TRE may negatively impact morning routines, social occasions, and HR-QoL outcomes. In contrast, individuals who habitually begin their daily routines late in the day and regularly partake in evening events may be more affected by earlier TRE protocols, in effect limiting the ability of TRE to positively impact HR-QoL outcomes. Anic et al^48^ also proposed this theory, as the authors suggested that improvements in HR-QoL may have been related to the flexibility of the TRE protocol, as participants were able to self-select mealtimes they preferred to omit and include. Thus, it could be suggested that the timing of the TRE protocol and its effects on an individual’s HR-QoL is based on the individual. As a result, participants may benefit from self-selecting the TRE protocol to ensure that the time and duration align with their daily routines.

Despite this theory, there were no distinct differences in HR-QoL outcomes between studies that provided participants with autonomy in the TRE protocol and those that did not.^32^^,^^34^^,^^41–48^ Nonetheless, the possibility that autonomy may play a role in improving HR-QoL outcomes cannot be dismissed. The comparability of HR-QoL outcomes based on autonomy was limited because of the fact that a larger number of studies provided participants with a choice (n = 7)^32^^,^^43–48^ compared with those that did not (n = 3).^34^^,^^41^^,^^42^ Additionally, research shows that autonomy effectively enhances a participant’s adherence to dietary interventions, including TRE.^17^^,^^73^ This has been attributed to participants feeling as though they have a say in the research process and still have ownership of their actions and mitigates the likelihood that the TRE protocol interferes with social eating and drinking occasions,^17^ in effect promoting greater adherence and increasing the possibility that TRE elicits more significant improvements in HR-QoL and metabolic and health outcomes. Nevertheless, due to the limited available literature and significant variability between studies, there appears to be no clear relationship between autonomy, adherence, and HR-QoL outcomes, and greater improvements in HR-QoL also did not appear to elicit greater adherence to TRE interventions and vice versa.^32^^,^^34^^,^^41–48^ Thus, the relationship between autonomy, adherence, and HR-QoL warrants further research.

### Strengths and limitations

Strengths of this study include that this was the first systematic review to gather and compare the effects of TRE on HR-QoL in adults to understand the current literature in this area. Additionally, an extensive literature search was conducted to gather potentially eligible studies, and screening was done by two researchers. In effect, this reduced the likelihood of missing eligible studies and selection bias. Moreover, a quality analysis was also done on each paper, which revealed that the risk of bias was low in most included studies. Finally, this quality analysis was cross-checked via a second researcher to reduce the risk of bias.

In contrast, there were various limitations that should be noted when interpreting the outcomes and theories proposed in this systematic review. This includes the small number of studies included, and significant heterogeneity between studies, including population, age, health status, HR-QoL measures, TRE protocol, duration, and co-interventions. In effect, the lack of homogeneity made it difficult to analyze and compare HR-QoL outcomes in TRE studies. The small sample of studies limits the ability to identify the true impact of TRE on HR-QoL and distinguish key factors that may influence HR-QoL outcomes in TRE interventions. The lack of research eligible for inclusion in this review was due to the limited number of TRE studies that measured HR-QoL as an outcome. More specifically, 77 TRE studies were excluded because they did not measure HR-QoL. Additionally, some studies included may have been underpowered, thus unable to elicit statistically meaningful changes in HR-QoL. Three of the studies included were deemed low-quality research papers, and thus were subject to greater bias; their results need to be interpreted with caution. In effect, this may have skewed the interpretation of HR-QoL outcomes. The inclusion of low-quality research papers may have also been because this review included a diverse range of study designs—that is, RCTs and pre-post pilot and feasibility studies.

## CONCLUSION

Overall, the findings from studies investigating the impact of TRE on HR-QoL among adults indicate that the current evidence is inconclusive. Nonetheless, TRE interventions did elicit improvements in overall, or some aspects of, HR-QoL in most studies, while TRE also did not appear to negatively impact HR-QoL in participants. Therefore, TRE may be a feasible dietary intervention used in practice to improve body weight and associated metabolic outcomes, without adversely affecting HR-QoL. Finally, it is also plausible that HR-QoL outcomes in TRE interventions may be influenced by other factors, including duration of adherence, autonomy, TRE protocol, improved body weight, population health status, and age. However, the true effect of these parameters on HR-QoL, and the overall effect of TRE on HR-QoL, cannot be concluded due to the current limited body of research and significant heterogeneity among the included studies. Therefore, further research investigating the impact of TRE on HR-QoL in adults is required. These studies should be high-quality research designs that include population samples with enough power to elicit meaningful results. Future research should also investigate HR-QoL outcomes after longer TRE interventions in varying populations (with and without chronic disease), and how changes in HR-QoL may be influenced by additional study outcomes (eg, weight loss), autonomy, and varying TRE protocols. As a result, this will give clinicians and researchers a greater understanding of barriers and facilitators to TRE interventions, and their effectiveness in practice.

## Supplementary Material

nuae044_Supplementary_Data

## Supporting Information

The following Supporting Information is available through the online version of this article at the publisher’s website.

Appendix S1. **PRISMA 2020 checklist**
